# Influenza Pneumonia Surveillance among Hospitalized Adults May Underestimate the Burden of Severe Influenza Disease

**DOI:** 10.1371/journal.pone.0113903

**Published:** 2014-11-25

**Authors:** Justin R. Ortiz, Kathleen M. Neuzil, Colin R. Cooke, Moni B. Neradilek, Christopher H. Goss, David K. Shay

**Affiliations:** 1 Department of Medicine, University of Washington, Seattle, Washington, United States of America; 2 Department of Global Health, University of Washington, Seattle, Washington, United States of America; 3 Vaccine Access and Delivery Global Program, PATH, Seattle, Washington, United States of America; 4 Department of Medicine, University of Michigan, Ann Arbor, Michigan, United States of America; 5 The Mountain-Whisper-Light Statistics, Seattle, Washington, United States of America; 6 Influenza Division, Centers for Disease Control and Prevention, Atlanta, Georgia, United States of America; Arizona State University, United States of America

## Abstract

**Background:**

Studies seeking to estimate the burden of influenza among hospitalized adults often use case definitions that require presence of pneumonia. The goal of this study was to assess the extent to which restricting influenza testing to adults hospitalized with pneumonia could underestimate the total burden of hospitalized influenza disease.

**Methods:**

We conducted a modelling study using the complete State Inpatient Databases from Arizona, California, and Washington and regional influenza surveillance data acquired from CDC from January 2003 through March 2009. The exposures of interest were positive laboratory tests for influenza A (H1N1), influenza A (H3N2), and influenza B from two contiguous US Federal Regions encompassing the study area. We identified the two outcomes of interest by ICD-9-CM code: respiratory and circulatory hospitalizations, as well as critical illness hospitalizations (acute respiratory failure, severe sepsis, and in-hospital death). We linked the hospitalization datasets with the virus surveillance datasets by geographic region and month of hospitalization. We used negative binomial regression models to estimate the number of influenza-associated events for the outcomes of interest. We sub-categorized these events to include all outcomes with or without pneumonia diagnosis codes.

**Results:**

We estimated that there were 80,834 (95% CI 29,214–174,033) influenza-associated respiratory and circulatory hospitalizations and 26,760 (95% CI 14,541–47,464) influenza-associated critical illness hospitalizations. When a pneumonia diagnosis was excluded, the estimated number of influenza-associated respiratory and circulatory hospitalizations was 24,816 (95% CI 6,342–92,624). The estimated number of influenza-associated critical illness hospitalizations was 8,213 (95% CI 3,764–20,799). Around 30% of both influenza-associated respiratory and circulatory hospitalizations, as well as influenza-associated critical illness hospitalizations did not have pneumonia diagnosis codes.

**Conclusions:**

Surveillance studies which only consider hospitalizations that include a diagnosis of pneumonia may underestimate the total burden of influenza hospitalizations.

## Introduction

Policy makers who implement influenza vaccine programs or interventions to increase influenza vaccine coverage must first understand the epidemiology of influenza in the targeted population. For example, results of studies of severe influenza morbidity and mortality influenced the World Health Organization (WHO) to develop a policy to broaden influenza vaccination programs to cover pregnant women [1]. Similarly, epidemiologic data on the burden of pediatric influenza disease were instrumental in recent decisions by the United States and the United Kingdom to expand influenza vaccine recommendations to cover all children [1]–[3]. Most high-income, temperate countries have identified adults, particularly elderly adults, as being a high-risk group for influenza complications and death, and therefore a priority group for vaccine receipt [1], [2]. In the United States, over 23,000 adults, mostly elderly, are estimated to die from influenza and its complications yearly, though annual mortality estimates vary considerably from year to year [4]. Because little influenza surveillance is conducted in low-resource countries, it is unknown whether there is a similar disproportionate burden of adult influenza in these settings.

In an effort to standardize influenza surveillance and to increase influenza morbidity data collection from low resource settings, the WHO established guidelines to standardize influenza surveillance worldwide and developed tools to use such data to estimate the burden of severe influenza disease [5], [6]. The WHO initiative to standardize hospitalized influenza surveillance has focused on identification of patients with severe acute respiratory infections (SARI) in sentinel hospitals, with collection of epidemiologic data and clinical specimens for influenza-specific laboratory testing limited to these patients [6]. To date, published SARI surveillance studies use evidence of hospitalization for acute febrile, lower respiratory tract illness as the case definition for study entry and influenza testing [7]–[16]. However, influenza virus infection can precipitate a wide range of processes necessitating hospital care, and case definitions that require evidence of acute lower respiratory tract infection will overlook non-pneumonia complications of influenza, including exacerbations of asthma, chronic obstructive lung disease, and congestive heart failure [17].

The goal of this study was to assess the extent to which restricting influenza testing to adults hospitalized with pneumonia may underestimate the total burden of hospitalized influenza disease. We hypothesized that the burden of influenza disease was considerably greater than that captured by influenza pneumonia surveillance.

## Methods

### Study design

We conducted a modelling study to estimate the number of adult influenza-associated hospitalizations overall and among those without pneumonia diagnoses in Arizona, California, and Washington from January 2003 through March 2009. We used state hospitalization databases from the Healthcare Cost and Utilization Project (HCUP) and regional influenza surveillance data from the US Centers for Disease Control and Prevention (CDC) [18], [19]. Our outcomes of interest were respiratory and circulatory hospitalizations and critical illness hospitalizations defined using ICD-9-CM codes. Our exposures of interest were positive tests for influenza from regional surveillance. We linked the datasets by calendar month and geographic region. We then used negative-binomial regression models to estimate the number of influenza-associated outcomes with or without pneumonia diagnosis.

### Influenza surveillance dataset

Regional surveillance data for influenza A (H3N2), influenza A (H1N1), influenza B for US Federal Regions 9 (AZ, CA, HI, NV) and 10 (AK, ID, OR, WA) were obtained from the CDC [18]. We collapsed the week-level data by month to permit linkage to the hospitalization dataset. We defined the influenza surveillance year as July through June of the next calendar year in order to capture an entire influenza season annually. For unsubtyped influenza A viruses, we assumed that the subtypes were proportional to the total influenza A (H3N2) and influenza A (H1N1) viruses detected over the entire surveillance year [20], [21]. We next divided the total number of positive tests per influenza type and subtype by the total number of tests performed in the surveillance year to reduce possible bias associated with differences in surveillance testing over time [20], [21].

### Hospitalization dataset

We used the State Inpatient Database (SID) for Arizona, California, and Washington from the Healthcare Cost and Utilization Project (HCUP) [19]. The outcomes of interest were defined by ICD-9-CM diagnosis and procedure codes (Table S1). Respiratory and circulatory outcomes were defined as any hospitalizations with ICD-9-CM codes 390-519 [21], [22]. Critical illness hospitalizations were defined as any hospitalizations with ICD-9-CM diagnosis or procedure codes for acute respiratory failure (96.7 and any of 518.5, 518.81, or 518.82) [23], severe sepsis (995.92, 785.52, or any codes for infection and organ dysfunction) [24]–[26], or in-hospital death (HCUP variable died  = 1) [27]. We categorized the outcomes by five adult age groups: 18–49 years, 50–64 years, 65–74 years, 75–84 years, and 85 years and older, as well as by presence or absence of ICD-9-CM diagnoses for pneumonia (480–486) excluding concurrent influenza diagnosis codes (487).

### Statistical analysis

We used negative binomial regression models similar to those developed by CDC to estimate US influenza-associated hospitalizations [4], [21], [22]. We recently adapted this model to estimate the incidence of influenza-associated acute respiratory failure [20]. A detailed description of our statistical methods has been published [20].

Briefly, age group-specific negative binomial regression models were fit to monthly events in the three states of interest. Covariates for the standardized proportion of specimens testing positive each month for influenza A (H1N1), influenza A (H3N2), and influenza B in the two US Federal Regions were included in the models. Additional covariates accounted for seasonal trend, secular trend, and state. Using the fitted (baseline) model, we then calculated the number of the influenza-associated outcomes for each influenza type/subtype as the difference between the outcomes estimated from the original data and the outcomes with the given influenza type term set to zero (the difference was set to zero when negative). This method defines the difference between predicted and baseline events as influenza-associated events. The baseline accounted for the seasonality of non-influenza associated events, some of which may be associated with other respiratory pathogens and some of which are likely due to non-infectious causes. Only a small fraction of events occurring during the winter are thus attributed to influenza infections. The modeled monthly outcomes were summed for each viral covariate across the study period and states for each outcome and age category. We calculated 95% confidence intervals for influenza-associated events using the non-parametric bootstrap [28].

This study received exempt review status from the Human Subjects Division at the University of Washington (#HSD No. 36982). No consent was obtained for the use of patient-level data. The records analyzed were anonymized and de-identified prior to analysis. Analyses were performed with SAS (version 9.3; Cary, NC) and R (version 3.0.1; Austria, Vienna) statistical software.

## Results

During the study period, there were 28,176,969 hospitalizations in the three states; 16,044,193 (57% of total) hospitalizations were for respiratory or circulatory outcomes, while 2,066,072 (7% of total) were for critical illness outcomes (Table 1). Almost all of the critical illness hospitalizations (94.1%) had ICD-9 codes for respiratory and circulatory diagnoses, while few respiratory and circulatory diagnoses had ICD-9 codes for critical illness (12.1%). Using multivariate binomial regression models, we estimated that there were 80,834 (95% CI 29,214–174,033) influenza-associated respiratory or circulatory hospitalizations and 26,760 (95% CI 14,541–47,464) influenza-associated critical illness hospitalizations (Table 1). We estimated annual incidence estimates of influenza-associated respiratory and circulatory hospitalizations for 18–49 years (14.8 per 100,000 person years), 50–64 years (24.2 per 100,000 person years, 65–74 years (40.3 per 100,000 person years), 75–84 years (144.9 per 100,000 person years), and ≥85 years (531.3 per 100,000 person years) (Table S2).

**Table 1:** Estimated Influenza Associated Hospitalizations^1^ with and without Pneumonia by Clinical Outcome, AZ, CA, & WA, January 2003 through March 2009.

Age (years)	Total Influenza-Associated respiratory & circulatory Hospitalizations^2^ n, (95% CI)		Influenza-Associated respiratory & circulatory Hospitalizations^2^ *without Pneumonia* ^3^ n, (95% CI)		Total Influenza-Associated critical illness Hospitalizations^2^ n, (95% CI)		Influenza-Associated critical illness Hospitalizations^2^ *without Pneumonia* ^3^ n, (95% CI)	
18–49	20,837	(6,986–42,870)	8,320	(402–25,377)	3,161	(1,108–6,491)	1,143	(122–3,536)
50–64	11,842	(0–32,712)	3,990	(0–20,081)	4,986	(2,147–10,257)	1,927	(378–5,367)
65–74	7,012	(0–31,160)	1,193	(0–16,963)	5,022	(2,213–9,253)	1,644	(356–4,232)
75–84	17,197	(2,021–46,251)	2,507	(0–22,850)	6,553	(3,039–12,759)	1,524	(81–5,489)
85+	23,945	(9,770–42,708)	8,806	(1,121–22,373)	7,038	(3,533–12,050)	1,975	(812–4,562)
Overall	80,834	(29,214–174,033)	24,816	(6,342–92,624)	26,760	(14,541–47,464)	8,213	(3,764–20,799)

1. Outcome definitions are detailed in the Table S1. Respiratory and Circulatory Hospitalizations were defined by ICD-9-CM codes for hospitalizations with any respiratory or circulatory diagnoses. Critical illness Hospitalizations were defined by ICD-9-CM diagnosis and procedure codes for hospitalizations with acute respiratory failure, severe sepsis, or in-hospital death.

2. Total influenza-associated events and total influenza-associated events without pneumonia were estimated from negative binomial regression models.

3. Pneumonia defined by ICD-9-CM codes for pneumonia diagnosis codes excluding concurrent influenza diagnosis codes.

We repeated these analyses excluding outcomes that listed a diagnostic code for pneumonia. When a pneumonia diagnosis was excluded, the estimated number of influenza-associated respiratory or circulatory hospitalizations was 24,816 (95% CI 6,342–92,624). The estimated number of influenza-associated critical illness hospitalizations excluding pneumonia diagnoses was 8,213 (95% CI 3,764–20,799). These data suggest that 31% of both the total influenza-associated respiratory and circulatory hospitalizations and the total influenza-associated critical illness hospitalizations do not have pneumonia diagnoses and may not be captured by hospital-based influenza pneumonia surveillance.

Within adult age groups, we estimate that requiring pneumonia diagnoses to define influenza-associated events would exclude from 17.0% (65 to 74 years) to 39.9% (18 to 49 years) of total influenza-associated respiratory and circulatory hospitalizations. For influenza-associated critical illness hospitalizations, requiring a pneumonia diagnoses would exclude from 23.3% (75 to 84 years) to 38.7% (50 to 64 years) of total events.

## Conclusions

Our study found that disease modelling approaches will estimate 50% fewer cases of influenza-associated hospitalizations if pneumonia ICD-9-CM codes are required as compared to more sensitive respiratory or circulatory ICD-9-CM codes. This study has implications for influenza disease surveillance in hospitals and for estimates of influenza disease burden. If adult influenza surveillance is limited to patients with pneumonia, our data suggest that a substantial proportion of influenza-associated hospitalizations may be missed. Such underestimates might affect the perceived value or cost-effectiveness of influenza vaccination, particularly in countries with only limited influenza surveillance data.

Non-pneumonia complications of influenza virus infection are more likely to occur in older adults with chronic comorbid conditions [29]. The United States has an older population with a higher prevalence of chronic underlying medical conditions than typical low- or middle-income countries [30]. These demographic differences suggest that influenza-pneumonia case definitions may be more sensitive for all-cause influenza disease in different socio-demographic settings. A recent study from South Africa estimates that influenza associated deaths in the elderly due to cerebrovascular disease, diabetes, or ischemic heart disease were higher than in the United States [31], and younger adults with AIDS in South Africa had elevated incidence of non-pneumonia influenza-attributable mortality [32]. With increasing survival and rising prevalence of non-communicable diseases throughout the world [30], influenza-associated exacerbations of underlying diseases will likely increase in importance.

Our study should be interpreted in the context of its limitations. The ideal study design to address our primary objective would be prospective hospital surveillance with sensitive specimen collection criteria for influenza testing and standardized radiographic assessment for pneumonia. Few such studies have been conducted, however, likely owing to the high costs of such research and the large sample sizes needed for high precision estimates, particularly among the critically ill. Owing to the nature of our modelling approach, caution should be taken with direct extrapolation of our results to the individual level. Because SID databases do not have results for microbiological investigations or particular therapies, we are unable to calculate the number of hospitalized influenza cases, necessitating our disease modeling approach. Similar methods have been used to estimate the burden of influenza disease in many countries [22], [33]–[37], and they are recommended by WHO to estimate influenza burden of disease when administrative hospitalization datasets and robust influenza surveillance data are available [5]. A recent study has validated our approach to estimating influenza-associated hospitalizations against prospective surveillance for laboratory-confirmed influenza in children [38], but there are no such validation studies in adults. The event estimates assumed that influenza types/subtype effects are non-negative and that virus activity is independent by type/subtype. If either of these two assumptions were violated, we may have over-estimated the total number of influenza-associated events. We used administrative codes to identify persons with acute respiratory failure, pneumonia, orsevere sepsis. While we used definitions of these outcomes from previous clinical epidemiology research, only pneumonia and severe sepsis have been validated against medical record review [25], [26], [39], [40].

Influenza vaccines are safe and effective at preventing influenza disease [2]. Vaccination would likely have the highest impact in settings where influenza morbidity is highest. Many countries will want local or regional burden of disease data to inform estimates of vaccine program impact in order to decide among competing public health priorities. Our study suggests that there may be a large proportion of unmeasured severe influenza disease in the influenza literature which must be considered in vaccine program impact calculations.

## Supporting Information

Table S1 **Critical Illness Outcome Definitions.**
(DOCX)

Table S2 **Estimated Incidence of Influenza-Associated Respiratory and Circulatory Hospitalizations per 100,000 person-years from Arizona, California, and Washington, January 2003 through March 2009.**
(DOCX)
