# Supplementary material for: Influenza Pneumonia Surveillance among Hospitalized Adults May Underestimate the Burden of Severe Influenza Disease
Source: PLoS One. 2014 Nov 25;9(11):e113903. doi: 10.1371/journal.pone.0113903 (PMC4244176; doi:10.1371/journal.pone.0113903)
Supplement: Table S1 — Critical Illness Outcome Definitions. (DOCX) [file pone.0113903.s001.docx]

**Table S1. Critical Illness Outcome Definitions**

1. **Acute Respiratory Failure** (Behrendt 2000 and Ortiz 2013)

- Any code for acute respiratory distress or failure (ICD-9-CM 518.5, 518.81, or 518.82); AND
- Has a procedure code for continuous mechanical ventilation (ICD-9-CM 96.7)

1. **Critical Illness** (Seymour 2010)

- All hospitalizations that meet any of the outcome definitions for Severe Sepsis, Acute respiratory Failure, or In-Hospital Death.

1. **Influenza** (CDC 2010 and Zhou 2012)

- All hospitalizations with ICD-9-CM Codes 487.xx

1. **In-Hospital Death** (Healthcare Cost and Utilization Project 2003-2009)

- SID variable died=1

1. **Pneumonia** (Zhou 2012)

- All hospitalizations with ICD-9-CM codes 480-486

1. **Respiratory and Circulatory Hospitalizations** (Zhou 2012)

- All hospitalizations with ICD-9-CM codes 390-519

1. **Sepsis or Infection** (Gaieski 2013, Iwashyna 2012, and Seymour 2012)

- All hospitalizations with ICD-9-CM Codes 995.91 (sepsis); OR
- All acute care hospitalizations with ICD-9-CM Codes Used to Identify a Bacterial or Fungal Infection 001, Cholera; 002, Typhoid/paratyphoid fever; 003, Other salmonella infection; 004, Shigellosis; 005, Other food poisoning; 008, Intestinal infection not otherwise classiﬁed; 009, Ill-deﬁned intestinal infection; 010, Primary tuberculosis infection; 011, Pulmonary tuberculosis; 012, Other respiratory tuberculosis; 013, Central nervous system tuberculosis; 014, Intestinal tuberculosis; 015, Tuberculosis of bone and joint; 016, Genitourinary tuberculosis; 017, Tuberculosis not otherwise classiﬁed; 018, Miliary tuberculosis; 020, Plague; 021, Tularemia; 022, Anthrax; 023, Brucellosis; 024, Glanders; 025, Melioidosis; 026, Rat-bite fever; 027, Other bacterial zoonoses; 030, Leprosy; 031, Other mycobacterial disease; 032, Diphtheria; 033, Whooping cough; 034, Streptococcal throat/scarlet fever; 035, Erysipelas; 036, Meningococcal infection; 037, Tetanus; 038, Septicemia; 039, Actinomycotic infections; 040, Other bacterial diseases; 041, Bacterial infection in other diseases not otherwise speciﬁed; 090, Congenital syphilis; 091, Early symptomatic syphilis; 092, Early syphilis latent; 093, Cardiovascular syphilis; 094, Neurosyphilis; 095, Other late symptomatic syphilis; 096, Late syphilis latent; 097, Other and unspeciﬁed syphilis; 098, Gonococcal infections; 100, Leptospirosis; 101, Vincent’s angina; 102, Yaws; 103, Pinta; 104, Other spirochetal infection; 110, Dermatophytosis; 111, Dermatomycosis not otherwise classiﬁed or speciﬁed; 112, Candidiasis; 114, Coccidioidomycosis; 115, Histoplasmosis; 116, Blastomycotic infection; 117, Other mycoses; 118, Opportunistic mycoses; 320, Bacterial meningitis; 322, Meningitis, unspeciﬁed; 324, Central nervous system abscess; 325, Phlebitis of intracranial sinus; 420, Acute pericarditis; 421, Acute or subacute endocarditis; 451, Thrombophlebitis; 461, Acute sinusitis; 462, Acute pharyngitis; 463, Acute tonsillitis; 464, Acute laryngitis/tracheitis; 465, Acute upper respiratory infection of multiple sites/not otherwise speciﬁed; 481, Pneumococcal pneumonia; 482, Other bacterial pneumonia; 485, Bronchopneumonia with organism not otherwise speciﬁed; 486, Pneumonia, organism not otherwise speciﬁed; 491.21, Acute exacerbation of obstructive chronic bronchitis; 494, Bronchiectasis; 510, Empyema; 513, Lung/mediastinum abscess; 540, Acute appendicitis; 541, Appendicitis not otherwise speciﬁed; 542, Other appendicitis; 562.01, Diverticulitis of small intestine without hemorrhage; 562.03, Diverticulitis of small intestine with hemorrhage; 562.11, Diverticulitis of colon without hemorrhage; 562.13, Diverticulitis of colon with hemorrhage; 566, Anal and rectal abscess; 567, Peritonitis; 569.5, Intestinal abscess; 569.83, Perforation of intestine; 572.0, Abscess of liver; 572.1, Portal pyemia; 575.0, Acute cholecystitis; 590, Kidney infection; 597, Urethritis/ urethral syndrome; 599.0, Urinary tract infection not otherwise speciﬁed; 601, Prostatic inﬂammation; 614, Female pel vic inﬂammation disease; 615, Uterine inﬂammatory disease; 616, Other female genital inﬂammation; 681, Cellulitis, ﬁnger/toe; 682, Other cellulitis or abscess; 683, Acute lymphadenitis; 686, Other local skin infection; 711.0, Pyogenic arthritis; 730, Osteomyelitis; 790.7, Bacteremia; 996.6, Infection or inﬂammation of device/graft; 998.5, Postoperative infection; 999.3, Infectious complication of medical care not otherwise classiﬁed. Where 3or 4-digit codes are listed, all associated subcodes will be included.

1. **Severe Sepsis or Infection with Organ Dysfunction** (Gaieski 2013, Iwashyna 2012, and Seymour 2012)

- All hospitalizations with ICD-9-CM Codes 995.92 (severe sepsis) OR 785.52 (septic shock); OR
- All sepsis hospitalizations (see above for corresponding definition) that also have a diagnosis of acute organ dysfunction, including shock without trauma, 785.5; Hypotension, 458; Mechanical ventilation, 96.7; Encephalopathy, 348.3; Transient organic psychosis, 293; Anoxic brain damage, 348.1; Secondary thrombocytopenia, 287.4; Thrombocytopenia, unspeciﬁed, 287.5; Other/unspeciﬁed coagulation defect, 286.9; deﬁbrination syndrome, 286.6; Acute and subacute necrosis of liver, 570; Hepatic infarction, 573.4; Acute renal failure, 584.

**REFERENCES:**

- Behrendt CE. Acute respiratory failure in the united states: Incidence and 31-day survival. Chest 2000;118:1100-1105.
- Centers for Disease Control and Prevention. Estimates of deaths associated with seasonal influenza --- united states, 1976-2007. MMWR Morbidity and mortality weekly report 2010;59:1057-1062.
- Gaieski DF, Edwards JM, Kallan MJ, Carr BG. Benchmarking the incidence and mortality of severe sepsis in the united states. critical care medicine 2013;41:1167-1174.
- Healthcare Cost and Utilization Project (HCUP). HCUP State Inpatient Databases (SID). Rockville, MD: Agency for Healthcare Research and Quality; 2003-2009.
- Iwashyna TJ, Odden A, Rohde J, Bonham C, Kuhn L, Malani P, Chen L, Flanders S. Identifying patients with severe sepsis using administrative claims: Patient-level validation of the angus implementation of the international consensus conference definition of severe sepsis. Medical Care 2012.
- Seymour CW, Rea TD, Kahn JM, Walkey AJ, Yealy DM, Angus DC. Severe sepsis in pre-hospital emergency care: Analysis of incidence, care, and outcome. American Journal of respiratory and critical Care Medicine 2012;186:1264-1271.
- Seymour CW, Kahn JM, Cooke CR, Watkins TR, Heckbert SR, Rea TD. Prediction of critical illness during out-of-hospital emergency care. JAMA : the journal of the American Medical Association 2010;304:747-754.
- Ortiz JR, Neuzil KM, Rue TC, Zhou H, Shay DK, Cheng PY, Cooke CR, Goss CH. Population-based incidence estimates of influenza-associated respiratory failure hospitalizations, 2003 - 2009. American Journal of respiratory and critical Care Medicine 2013.
- Zhou H, Thompson WW, Viboud CG, Ringholz CM, Cheng P-Y, Steiner C, Abedi GR, Anderson LJ, Brammer L, Shay DK. Hospitalizations associated with influenza and respiratory syncytial virus in the united states, 1993–2008. Clinical Infectious Diseases 2012;54:1427-1436.
