# Supplementary material for: Influenza Pneumonia Surveillance among Hospitalized Adults May Underestimate the Burden of Severe Influenza Disease
Source: PLoS One. 2014 Nov 25;9(11):e113903. doi: 10.1371/journal.pone.0113903 (PMC4244176; doi:10.1371/journal.pone.0113903)
Supplement: Table S2 — Estimated Incidence of Influenza-Associated Respiratory and Circulatory Hospitalizations per 100,000 person-years from Arizona, California, and Washington, January 2003 through March 2009. (DOCX) [file pone.0113903.s002.docx]

**Table S2. Estimated Incidence of Influenza-Associated Respiratory and Circulatory Hospitalizations per 100,000 person-years from Arizona, California, and Washington, January 2003 through March 2009**

| Age Group (years) | Total Respiratory  and Circulatory Hospitalizations | Person-Years | Influenza-Associated Respiratory and Circulatory Hospitalizations | Influenza-Associated Respiratory and Circulatory Hospitalization Incidence Rate per 100,000 Person-Years |
| --- | --- | --- | --- | --- |
| 18-49 | 2,874,738 | 140,843,294.8 | 20,822 | 14.8 |
| 50-64 | 3,960,137 | 48,957,998.5 | 11,834 | 24.2 |
| 65-74 | 3,268,791 | 17,372,578.0 | 7,007 | 40.3 |
| 75-84 | 3,813,526 | 11,864,962.5 | 17,191 | 144.9 |
| ≥85 | 2,127,001 | 4,505,319.3 | 23,939 | 531.3 |
| All | 16,044,193 | 223,544,153.0 | 80,793 | 36.1 |

Note: We have previously estimated the incidence of influenza-associated critical illness hospitalizations from the same dataset (Ortiz JR, Neuzil KM, Shay DK, Rue TC, Neradilek MB, et al. (2014) The burden of influenza-associated critical illness hospitalizations. Crit Care Med. 2014 Nov;42(11):2325-32.)
